# Chimpanzees and Bonobos Exhibit Emotional Responses to Decision Outcomes

**DOI:** 10.1371/journal.pone.0063058

**Published:** 2013-05-29

**Authors:** Alexandra G. Rosati, Brian Hare

**Affiliations:** Department of Evolutionary Anthropology, Center for Cognitive Neuroscience, Duke University, Durham, North Carolina, United States of America; University of Bologna, Italy

## Abstract

The interface between cognition, emotion, and motivation is thought to be of central importance in understanding complex cognitive functions such as decision-making and executive control in humans. Although nonhuman apes have complex repertoires of emotional expression, little is known about the role of affective processes in ape decision-making. To illuminate the evolutionary origins of human-like patterns of choice, we investigated decision-making in humans' closest phylogenetic relatives, chimpanzees (*Pan troglodytes*) and bonobos (*Pan paniscus*). In two studies, we examined these species' temporal and risk preferences, and assessed whether apes show emotional and motivational responses in decision-making contexts. We find that (1) chimpanzees are more patient and more risk-prone than are bonobos, (2) both species exhibit affective and motivational responses following the outcomes of their decisions, and (3) some emotional and motivational responses map onto species-level and individual-differences in decision-making. These results indicate that apes do exhibit emotional responses to decision-making, like humans. We explore the hypothesis that affective and motivational biases may underlie the psychological mechanisms supporting value-based preferences in these species.

## Introduction

Emotion and motivation are crucial in shaping human behavior, and the interaction between emotion, motivation, and cognition is a major topic in the human cognitive sciences. This increased interest in the role of emotion in complex behaviors is especially apparent in the study of decision-making, which has revealed that emotions play an important role in human choice processes [1]. In contrast, studies of animal decision-making have made great strides in illuminating the cognitive bases of choice [2] and how value is represented in the brain [3], [4]. However, it is currently unclear whether emotional responses are involved in nonhuman decision-making. Indeed, animals are thought to lack some relevant processes [5]. Thus, one possibility is that differences in decision-making and executive functioning between humans and other animals reflect differences in the emotional and motivational mechanisms supporting complex choice behavior. However, current evidence indicates that a wide range of taxa show clear behavioral, physiological, and neurobiological signs of emotional processes [6], especially for emotions such as fear and anxiety [7], [8]. Furthermore, some recent evidence suggests that a variety of nonhuman taxa exhibit emotional influences on perceptual judgments much like humans [9], [10]. Finally, nonhuman apes such as chimpanzees exhibit rich repertories of emotional expression [11], and therefore may experience some of the same emotions that are important in human decision-making. In the current studies, we therefore examine our closest living phylogenetic relatives – chimpanzees (*Pan troglodytes*) and bonobos (*Pan paniscus*) – in order to illuminate the origins of human decision-making.

Extending a comparative approach to the study of emotion, motivation, and cognition highlights the importance of an evolutionary or functional perspective towards these psychological processes (e.g., [12]). In humans, studies of emotion have often focused on either identifying basic types of discrete emotions [13], [14], or identifying the processes by which emotions arise (e.g., appraisal theories; [15], [16]). Cutting across approaches, however, are theories suggesting that the function of emotions is related to how they impact motivation, or desire to act. That is, emotions appear to allow the flexible generation of appropriate behavioral responses tailored to the current set of circumstances being experienced [17]. A broad stance is therefore that emotions are positive or negative states elicited by events in the environment [18], and motivation is a behavioral ‘drive’ to do something in response to these experiences (such as approach or withdraw). Thus, emotion and motivation are closely linked, with emotion focusing on how an individual assesses a given situation, and motivation focusing on how this assessment influences that individual's desire to act [19], [20]. For the purposes of the present studies with apes, we therefore use emotions to refer broadly to positive or negative states elicited by the evaluation of rewards or punishments, and motivation to refer to an individual's drive to acquire rewards or avoid punishments.

Chimpanzees and bonobos are an interesting test case for this comparative approach, as previous studies indicated that chimpanzees and bonobos differ in their decision-making preferences. When making temporal choices involving tradeoffs between reward and delays, chimpanzees are more willing wait to acquire larger rewards than are bonobos [21]. Similarly, when faced with risky choices involving variability in reward payoffs, chimpanzees are more willing to accept risk to acquire high-value payoffs [22], [23], [24]. However, the mechanistic basis for these differences in preferences is currently unclear. That is, from a psychological perspective, why do chimpanzees and bonobos choose differently when faced with the same problem? Given the importance of emotions in human decision-making, one possibility is that these species' different patterns of choice relate to differences in emotional processes. From an ultimate perspective, altering emotion and motivation may therefore be an important evolutionary pathway for generating different behavioral strategies across species.

There is clear evidence that emotions are important in shaping decisions both about risk and time in humans. For example, when making decisions under risk, people experience negative states such as disappointment or regret as a consequence of unfavorable outcomes [25], [26]. Furthermore, people anticipate that they will experience such emotions, and take this possibility into account when making decisions [27], [28], [29], [30], [31], [32], [33]. These emotional processes play a causal role in decision-making, as it is possible to shift preferences by experimentally manipulating states of mood or stress [34], [35], [36], [37], and individuals with brain lesions that impact emotional processes show altered patterns of choice [38], [39], [40]. Other types of decisions, such as intertemporal choices, also involve emotional processes [41]. While many human temporal choice experiments involve question-based measures that are not applicable to nonverbal animals, there is also some relevant data from experiential-based tasks. First, in terms of responses to waiting, some research suggests that people prefer more immediate outcomes when waiting is unpleasant or perceived as being especially costly, [42], [43], [44]. Delay of gratification studies further suggest that individuals who experience more negative emotions such as frustration while waiting will be less inclined to choose delayed payoffs [45], [46]. Finally, as with risk preferences, trait differences in mood may contribute to individual differences in intertemporal choice [47].

Overall, these studies suggest that interactions between emotion and decision-making in humans are quite complex: emotions can influence preferences via several different pathways, including emotional responses to decision outcomes, as well as the *anticipation* of such emotional experiences prior to even making a choice. Consequently, an important first step in determining whether similar affective processes also play a role in ape decision-making is to assess whether apes even exhibit emotional responses in these types of contexts. Apes produce complex emotional signals that exhibit strong homologies with human expressions [48], [49], [50], [51]. Experimental studies also indicate that apes perceive and categorize emotional displays [52], [53], [54], [55], show memory biases towards emotional stimuli [56], and show greater interest in viewing emotional scenes [57] – much like humans. However, these studies have generally focused on the behaviors that apes exhibit in naturalistic social contexts, or on their responses to conspecific interactions (e.g., [58]). Consequently, it is unknown if apes would exhibit similar responses in the types of economic decision-making contexts explored here.

In the current experiments, we aimed to examine whether chimpanzees and bonobos exhibited emotional responses following the outcomes of their decisions. We tested a sample of semi-free ranging, wild-born chimpanzees and bonobos on an intertemporal choice task and a risky choice task. In both tasks we then measured whether apes exhibited a suite of behavioral indices of affective state in response to their decision's outcome. Given that various states with negative valence have strong influences on human decision-making [26], [35], [37], [59], [60], here we focus on negative responses in the apes. First, we coded negative emotional vocalizations, focusing on pout moans, whimpers, and screams following established ethograms in these species [61], [62]. These vocalizations are emotional signals that occur naturally in negative situations, and involve characteristic facial expressions [51]. Second, we coded scratching, which has been widely used as a measure of anxiety or stress in primates [63], [64]. Third, we coded banging, a type of tantrum that may reflect anger in chimpanzees [58]. We based this measure on previous use of knocking or manipulating an apparatus to indicate negative responses in apes [65]. Finally in both studies we assessed a behavioral index of reward motivation.

We used these measures to address two main questions. First, we examined whether apes exhibit different responses to different decision outcomes like humans. In particular, in the temporal task we examined whether apes exhibited these responses more often when waiting, and in the risk task we examined whether apes exhibited these affective reactions more often in response to unfavorable outcomes. Second, we examined whether individual- or species-level variation in responses mapped on to decision-making preferences. In particular, we expected that the bonobos would be less patient and more risk-averse than chimpanzees, following previous studies [21], [22], [23], [24], [66]. Given these different patterns of choice, we therefore predicted that the two species may differ in their emotional responses to decision outcomes. Specifically, we predicted that the bonobos would exhibit more negative responses when waiting in temporal choice task, and more negative responses after receiving less-preferred payoffs in the risky choice task. That is, one possible explanation for these species patterns of choice is that bonobos experience more negative emotional states in response to waiting or following undesired outcomes, and this drives them to exhibit less patience and more risk-aversion than chimpanzees.

## Study 1: Temporal Preferences

In the first experiment, chimpanzees and bonobos chose between a small reward (one piece of food) that was available immediately, and a larger reward (three pieces) that was only available after either a one or two minutes delay.

### Materials and Methods

#### Ethics statement

All behavioral studies were noninvasive. The studies had approval from the Institutional Animal Care and Use Committee of Duke University (protocol number A078-08-03) and strictly adhered to the legal requirements of the countries in which they were conducted. The chimpanzee research was carried out with permission from Tchimpounga Chimpanzee Sanctuary in Pointe Noire, Republic of Congo and the Ministry of Scientific Research and Technological Innovation in Republic of Congo (permit: 009/MRS/DGRST/DMAST). The bonobo research was carried out with permission from Lola ya Bonobo Sanctuary in Kinshasa, Democratic Republic of Congo and the Ministry of Research and the Ministry of Environment in the Democratic Republic of Congo (permit: MIN.RS/SG/004/2009). Animal husbandry and care practices at both locations complied with the Pan-African Sanctuary Alliance (PASA) Primate Veterinary Healthcare Manual, as well as the policies of Tchimpounga Chimpanzee Sanctuary and Lola ya Bonobo Sanctuary respectively. All apes at both sites were socially housed, and majority semi-free-ranged in large tracts of tropical forest during the day (5–40 ha across groups). In the evening, all apes spent the night in indoor dormitories (12–160 m^2^). Apes were tested individually in these familiar buildings. Following testing, most apes were released back with their larger social group outside. Apes had *ad libitum* access to water and were never food deprived for testing. In addition to the food the apes could eat in their forest enclosures, they were fed a variety of fruits and vegetables and other species-appropriate food two to four times daily. Subjects completed no more than one test session per day, and all tests were voluntary: if the ape stopped participating, the session was halted.

#### Subjects

We tested 38 semi-free ranging apes: 23 chimpanzees from Tchimpounga Chimpanzee Sanctuary in Pointe Noire, Republic of Congo (9 females and 14 males; average age 12 years; range 7–20 years) and 15 bonobos from Lola ya Bonobo Sanctuary in Kinshasa, Republic of Congo (3 females and 12 males; average age: 8 years; range 7–10 years). The majority of the apes were born in the wild and came to the sanctuary after being confiscated at an early age (∼2–3 years old) as a result of the bushmeat trade (see Table S1 for individual subject information). Previous work indicates that sanctuary apes are psychologically healthy relative to other captive populations [67]. The apes were naïve to temporal-choice tasks and were tested individually by an unfamiliar experimenter. Subjects completed no more than one test session per day. One additional chimpanzee and one additional bonobo began the study, but stopped participating for more than 3 days and therefore were excluded.

#### Quantity discrimination pretest

Prior to beginning the studies, all subjects completed a pretest to show they understood the basic setup and discriminated between the different amounts of food. In four initial e*xposure* trials, only one piece of food was available to confirm subjects would point to the food. Next, subjects completed six *number* trials in which they had to choose between one and three pieces of food (following the same basic procedure described below). Subjects had to choose the larger amount of food on five of six trials to meet criteria and proceed to the main task.

#### Temporal choice procedure

Each subject completed two conditions where they chose between a smaller, immediately available reward (one piece of food), and a larger, delayed reward (three pieces). In particular, in the *one-minute* condition the larger reward was available after one minute, whereas in the *two minute* condition the delay was two minutes; order was counterbalanced across subjects. We predicted that apes should be more willing to wait when the delay was shorter if they were sensitive to this variation in temporal costs. We manipulated the delay to receiving the food following previous comparative work with primates [21], [68], [69], [70], [71]. Models from behavioral ecology suggest the delay to receiving the food may be the most relevant interval from an evolutionary perspective [72].

For each condition, apes first completed an introductory session with 14 *exposure* trials where only one option (small, immediate or large, delayed) was available each trial to introduce subjects to the different rewards and delays. That is, apes experienced that they would have to wait to acquire the larger reward, but could receive the smaller reward immediately, prior to actually choosing between these options. They then completed a test session with 4 *exposure* trials (to remind them of the reward contingencies), followed by 10 *choice* trial*s* where subjects chose between the two options. The side assignment for the two options was counterbalanced and quasi-randomized (no more than three trials in a row with the same side assignment) within sessions. Both species made choices about a preferred food type (chimpanzees: banana slices; bonobos: apple pieces) based on a food preference test (as described in study 2).

In the sessions, the experimenter (E) and the ape sat across from each other at a table (80 cm wide, 40 cm deep, 50 cm tall) with a sliding top, separated by wire mesh or bars (see Figure S1 for photos of setup). E placed the rewards on the table behind an occluder (61.5 cm wide, 20 cm deep, 30.5 cm tall), with one option on each side of the table. At the start of each trial, E removed the occluder and subjects viewed the options for 3 seconds. E then pushed the table forward so apes could chose by pointing at one of the options. Depending on their choice, E then either gave the subject the chosen food immediately (small reward) or after the appropriate delay (large reward). In the latter case, E removed the forgone option from the table, looked down, and did not interact with the ape until the delay ended. There was a constant 30 s inter-trial interval (ITI) between trials (timed with a stopwatch), starting when subjects placed the last piece of food in their mouth. Apes had 20 s to choose; if they failed to choose in this time, the trial was repeated the end of the session. If apes did not participate for three trials the session was halted and then repeated the next day.

#### Data coding and analysis

To assess apes' reactions in the task, we coded whether apes exhibited negative affect in the 10 s immediately after their choice (thus equating the time during which apes could produce the behaviors after both types of choice). Specifically, we coded three types of target behaviors as described previously: 1) *negative emotional vocalizations*, focusing on pout moans, whimpers, and screams; 2) *scratching,* or whether the subject scratched their body or head with their nails; and 3) *banging*, or whether the subject forcefully hit the bars or mesh in front of the table with their hands or feet. As we only examined a short time frame (e.g., the 10 s period following choice) on each trial, we coded emotional responses as present or absent. Finally, as an index of motivation to acquire the food in the task, we measured *duration of stay* at the testing location on trials where subjects choose the larger, delayed reward. As apes could move freely about the testing room, we coded the length of continuous time that the subject sat at the table after making their choice before they walked away for the first time. This measure is similar to previous studies that have looked at effort or participation level in a task [65]. Apes had to be present at the testing location for behaviors to be coded.

Choices were coded live by the experimenter; a second coder blind to hypotheses coded 20% of trials from video with excellent reliability (Cohen's κ = 1.0). Vocalization, scratching, banging, and duration of stay were coded from videotape by a coder blind to the hypotheses; a second coded assess 20% of trials for reliability. For vocalizations, both individuals then coded all trials where vocalization had been identified to code whether apes specifically produced the three target negative vocalizations (screams, whimpers, or pout moans). Reliability was excellent for all measures (scratching: Cohen's κ = 0.93; negative vocalizations: κ = 0.91; banging: κ = 0.95; duration of stay: r_p_  = 0.984, p<0.001).

For data analysis, choice percentages were arc-sine square-root transformed to normalize the data. We used parametric statistics to analyze choice data; when assumptions of sphericity were violated, the Huynh-Feldt correction was used. We analyzed emotional responses in two ways. First, apes received a composite affect score indicating how many of responses (vocalizing, scratching, or banging) they made on each trial, following previous work collapsing across behavioral categories to assess overall level of response [65], [73], [74]. This score therefore assessed response intensity, with a 0 indicting that the ape exhibited no target behaviors, and a 3 indicating that they produced all three. We also analyzed each behavioral response separately. As emotional responses were not normally distributed, we used non-parametric statistics for those comparisons; when applicable, we report only results that meet significance with Bonferroni corrections to correct for multiple comparisons.

### Results and discussion

#### Quantity discrimination pretest

Subjects took between one and three days to meet the number trial criteria (chimpanzees: M = 1.48, SE  = 0.12; bonobos: M = 1.67, SE  = 0.18). The two species did not differ in how many days it took them to reach the criterion (t_36_  = 0.88, p = 0.39, n.s.), indicating similar numerical comprehension and preference for the larger amount in the two species.

#### Temporal choices

**Figure 1:**
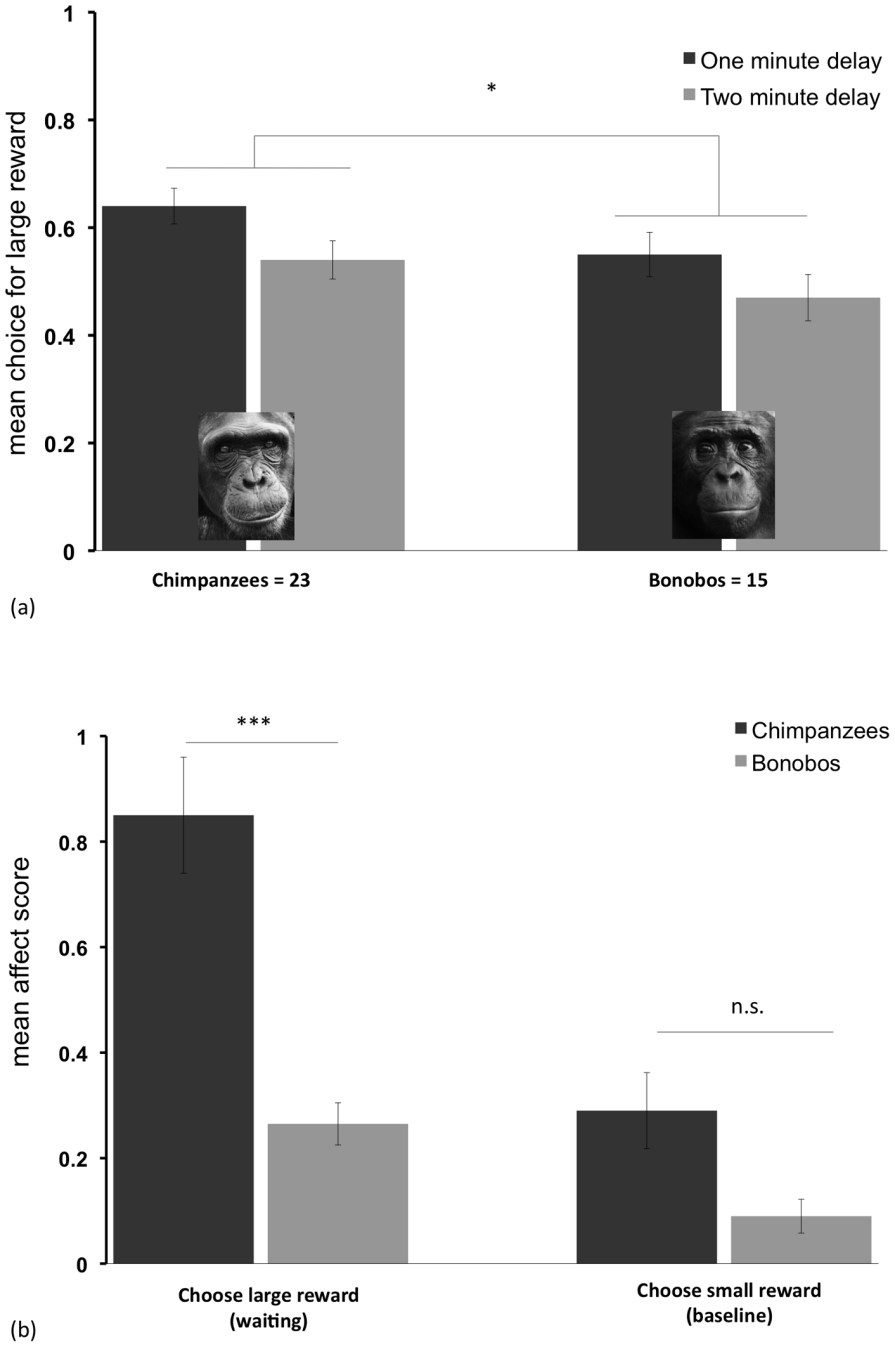
Temporal preferences and affective responses in study 1. a) Chimpanzees and bonobos chose between a smaller, immediate reward and a larger reward delayed by one or two minutes across conditions. b) Composite affect scores while waiting versus not waiting; a higher score indicates a more intense reaction with the production of more target behaviors (scratching, vocalizing, and banging). Error bars represent standard error of means.

Chimpanzees chose the large, delayed reward on 63.9±3.3% of trials in the one minute condition, and on 54.5±3.5% of trials in the two minute condition. Bonobos chose the large reward on 55.3±4.1% of trials in the one minute condition and 47.3±4.3% of trial in the two minute condition (see Figure 1A). A repeated-measures ANOVA revealed apes were more likely to chose to wait when faced with a one minute delay than a two minute delay (F_1,36_  = 5.36, p<0.05), indicating that apes were sensitive to the variation in delays and made tradeoffs between rewards and time costs. In addition, chimpanzees were more likely to wait overall.

(F_1,36_  = 4.22, p<0.05), with no significant interactions. A second analysis examining choices in the first and second half of the session showed that subject's preferences did not change within sessions (F_1,36_  = 1.43, p = 0.24, n.s.), with no species interaction, indicating no difference in rates of satiation or learning between the two species. Additional analyses indicated no impact of either age or sex on patterns of choice (see SI). Overall, these results indicate that chimpanzees were more willing to wait to receive larger rewards than bonobos, in line with previous comparisons of these species [21].

#### Emotional and motivational responses

We first examined apes' composite affect score to assess the intensity of the apes' emotional responses. Here a higher score indicated that apes exhibited more of the target negative reactions on that trial (see Figure 1B). Collapsing across conditions, we compared reactions when subjects were waiting after choosing the larger reward, versus when they chose the small reward as a behavioral baseline (see Videos S1 and S2). Whereas apes had low composite affect scores after they chose the small reward (mean affect score  = 0.21±0.05), scores were about three times higher when apes were waiting (mean score  = 0.61±0.09; Wilcoxon signed-rank test, n = 38, z = −5.08, T+  = 34, 1 tie, p<0.001). Moreover, apes were more likely to perform all three target behaviors more often when waiting after choosing the large, delayed reward (see Table 1 for means; n = 38 for all cases; *negative vocalization*: z = −4.02, T+  = 21, 17 ties, p<0.001; *scratching*: z = −2.93, T+  = 21, 8 ties, p<0.005; *banging*: z = −3.26, T+  = 16, 19 ties, p = 0.001). Overall, these results indicate that apes show negative affective responses when waiting. Importantly, we observed these reactions when apes freely chose to forgo the immediate reward to wait for larger payoffs.

**Table 1:** Affective responses in study 1 (temporal choice) and study 2 (risky choice).

	Temporal task (study 1)		Risk task (study 2)		
	Chose small (baseline)	Chose large (waiting)	Chose safe	Good outcome (chose risky)	Bad outcome (chose risky)
*Affect score*	0.21±0.05	0.61±0.09	0.10±0.03	0.08±0.02	0.29±0.06
*Scratching*	10.9± 2.7%	21.3±2.7%	6.8±1.4%	5.9±1.9%	16.4±3.7%
*Vocalizing*	7.7±3.5%	29.8±.5.4%	3.4±2.1%	1.1±0.1%	9.1±2.5%
*Banging*	2.8±1.3%	10.7±2.6%	0.5±0.5%	1.2±0.7%	4.3±1.9%
*Switching*	-	-	2.7±0.1%	3.5±1.5%	27.1±4.6%

In each task, we examined mean percent (± SE) of trials with target affective behaviors (*scratching, negative vocalizing*, and *banging*). *Affect score* indicates the mean composite score denoting response intensity across categories (for a given trial, 0 indicates subjects performed no target behaviors, whereas 3 indicates that they performed all three). In addition, we examined whether apes attempted to *switch* their choice following the reveal of the outcome in the risk task (outcomes were not hidden prior to choice in the temporal task).

We next examined whether affective responses mapped onto individual or species differences in temporal preferences. First, both species exhibited increases in the composite affect score while waiting, when examined separately (chimpanzees: n = 23, z = −4.04, T+  = 21, 1 tie, p<0.001; bonobos: n = 15, z = −3.18, T+  = 13, 0 ties, p<0.001). A comparison of the two species revealed that they showed similarly low affect scores when they chose the small reward (Mann-Whitney U: z = −1.69, N_1_ = 23, N_2_ = 15, p>0.10, n.s.), and exhibited no difference in the rate of any individual target behavior (p>0.12 for all cases.). However, a comparison of their responses while waiting for the large, delayed reward revealed that chimpanzees exhibited more intense responses (z = −3.65, N_1_ = 23, N_2_ = 15, p<0.001). Examining each target behavior individually revealed that the two species had similar rates of scratching and banging while waiting (p>0.24 for both behaviors, n.s.), but chimpanzees showed significantly more negative vocalizing when waiting (z = −3.88, N_1_ = 23, N_2_ = 15, p<0.001). In particular, whereas chimpanzees produced target vocalizations on 45.9±6.8% of trials when waiting, bonobos did so on only 5.1±3.3% of trials. Finally, we calculated a difference score for each subject as an individual index of affective response (average score while waiting, minus score after choosing the small reward as a baseline). Correlating this difference score with overall level of patience (e.g., the proportion of times that a given individual chose the larger, delayed reward) revealed no relationship in either species (Spearman's ρ, p>0.14, n.s. in both cases). Overall, these results indicated that while both species exhibited negative emotional responses to waiting, chimpanzees showed relatively more negative reactions than bonobos (particularly more negative vocalizing), contrary to our predictions. However, there was no relationship between individual differences in reactions and overall propensity in either species. This is possibly due to low individual variance in this task, as it involved few trials per subject.

Lastly, we compared *duration of stay* in the two species to assess motivation. Apes remained in front of the table an average of 44.3±2.7 s in the one minute condition, and an average of 57.8±6.5 s in the two minute condition (note that apes could wait a maximum of either 60 s or 120 s in the two conditions due to the task structure). A repeated-measures ANOVA revealed that apes remained longer at the table in the two-minute condition (F_1,36_  = 4.140, p<0.05). However, there was no effect of species (F_1,36_  = 2.091, p = 0.26, n.s.), nor any significant interactions. In addition, there was no relationship between duration of stay and overall temporal preferences in either species (p>0.70, n.s. in both cases). Thus, these results suggest that chimpanzees and bonobos were equally motivated to stay for food in the temporal task.

## Study 2: Risk Preferences

In the second study, we compared chimpanzees' and bonobos' willingness to accept variability in payoffs. Apes chose between a risky option that provided either a good (preferred) or a bad (non-preferred) food outcome with equal probability, versus a safe option that always delivered an intermediately-preferred food type (following the general procedure used in previous studies; [24], [66]). In addition, we varied the value of the safe option across trials to ensure that apes modulated their choices according to the relative value of the two options.

### Methods

#### Subjects

We tested 37 apes from the same populations as in study 1: 24 chimpanzees (10 females and 14 males; average age 12 years; range 7–20 years; 23 had participated in study 1) and 13 bonobos (3 females and 10 males; average age 8 years; range 7–10 years; 12 had participated in study 1; see Table S1). All individuals were naïve to the risk-choice task. As in study 1, subjects completed no more than one test session per day, and all tests were voluntary: if the ape stopped participating, the session was halted.

#### Food preference pretest

Subjects completed 20 *food preference* trials following the number pretest to determine appropriate foods for the risk task. Subjects from each species made pair-wise choices across those foods (chimpanzees: bread, banana, peanuts, papaya, cucumber; bonobos: apple, banana, papaya, peanuts, lettuce). Food types differed because of differences in availability at the two sanctuaries. On each trial, subjects saw two food options placed on opposite sides of the table for 4 s, and then E covered them with identical bowls. Apes chose between each possible pairing twice, in randomized order with side assignment counterbalanced.

#### Risky choice procedure

Each subject completed two conditions in counterbalanced order: a *low-variance* condition with only two possible risk outcomes, and a *high-variance* condition with four possible outcomes. In both conditions half the risk outcomes were good (preferred foods) and half were bad (non-preferred foods), but the number of total possible outcomes differed. In addition, we manipulated the value of the safe option by varying its number between one, three, and six pieces of food. Risk outcomes were randomly predetermined with good outcomes on 50% of trials in a session. The side assignment and safe value were counterbalanced and quasi-randomized within a session, with no more than three trials in a row with the same safe value or side-assignment. For each condition, subjects first completed an introductory session consisting of an initial 14 *exposure trials* (where only one option available at a time; eight risk option trials randomly intermixed with six safe option trials) followed by 8 *control trials* to assess task comprehension (see below). They then completed a *test session* with 18 *choice trials* and 4 randomly intermixed *control trials*.

In sessions, trials followed the basic procedure used previously in this task (e.g., [24], [66]; and see Figure S2 for photos of procedure). Subjects saw the experimenter (E) place the safe reward on one side of the table, and then cover it with an overturned bowl (17.5 cm in diameter, 5.5. cm tall). Next, subjects saw E place an identical – but empty – bowl on the table (the risk option). E then occluded the risk option with a small freestanding occluder (40.5 cm wide, 24 cm deep, 24 cm tall), and showed the subject the “risk outcome” container (a differently-colored bowl) containing the potential risk outcomes for that trial. In the low variance condition this consisted of one good outcome and one bad outcome (two potential outcomes total), whereas in the high-variance condition the container contained two good outcomes and two bad outcomes (four total). Behind the occluder, E then placed just one of these items under the risk bowl. That is, apes always only received only one of the possible set of outcomes they saw previously in the risk outcome container, without knowing beforehand which it would be. Finally, E touched both cups simultaneously while picking up the safe bowl to remind the subject of the safe option's value. Thus, subjects always knew what they would receive from the safe option, but did not know whether they would receive a desirable or undesirable outcome from the risky option. E then pushed the table forward for choice. As in study 1, there was a 30 s ITI between trials, starting when subjects put the last piece of food in their mouth. Subjects had 20 s to choose once the table was pushed forward; if they failed to choose in this time, the trial was repeated the end of the session. If apes did not participate for 3 trials the session was stopped and repeated the next day.

#### Control trials

Following previous studies using this basic task [24], [66], apes completed five types of controls across introductory and test sessions to confirm they understood the setup (see Figure S3 for diagram). In control trials, the number of possible outcomes in the risky outcome container was kept constant according to condition, and subjects always received only one of the possible outcomes from the risky option.

1. In *inhibition trials* (4 trials total–introductory sessions), the procedure was identical to normal choice trials, but in a final step the subject saw E removed the food from under the safe option bowl. If subjects could inhibit reaching for the last location where they saw food, as they observed that it had subsequently been removed, they should choose the risky option.
2. In *comprehension-1 trials* (6 trials – introductory sessions), the safe option provided two (in the low variance; or four in the high-variance) identical pieces of preferred food, and subjects saw two (or four in the high variance condition) pieces of the same food type in the risk outcome container. If subjects understood that they only received *one* of the potential risk outcomes, they should prefer the safe option as it would deliver *more* food – even though they initially saw the same amount of food associated with both the safe option and the risky outcome container.
3. In *comprehension-2 trials* (6 trials – introductory sessions), the safe option provided a small piece of a preferred food, and the risky outcome container contained two (or four in the high-variance condition) larger pieces of the preferred food. If subjects actively compared the potential rewards they could receive from the safe and risky options, they should prefer the risky option because it will deliver a *bigger* piece of preferred food.
4. In *attention-1 trials* (4 trials – test sessions), the safe option provided one piece of a preferred food and subjects saw two (or four in the high-variance condition) pieces of a non-preferred food in the risky outcome container. Subjects should choose the safe option because it delivers a more-preferred food type.
5. In *attention-2 trials* (4 trials – test sessions), the safe option was the non-preferred food, and the risk outcome bowl contained two (or four in the high-variance condition) pieces of preferred food. Subjects should choose the risky option because it delivered a more-preferred food type. Taken together, the two attention control trial types thus ensured that apes attended to the available options on a trial-by-trial basis in test sessions.

#### Data coding and analysis

As in study 1, choices were coded live by the experimenter; a second coder blind to hypotheses coded 20% of trials from video with excellent reliability (Cohen's κ = 1.0). We coded emotional responses using the methods described in study 1 (*negative vocalizations, scratching*, and *banging*), here in the 10 s after the experimenter revealed the choice outcome. In addition, we coded whether the ape attempted to *switch* their choice in the 5 s after the outcome was revealed – that is, whether they tried to point at the forgone option immediately after seeing what they had had actually received from their choice. Reliability with a second coder was excellent (*scratching*: κ = 0.97; *negative vocalizing*: κ = 0.93; *banging*: κ = 1.0; *switching:* κ = 0.96). Finally, as an index of motivation we examined reward sensitivity, or how previous trial outcomes impacted current trial choice. Analysis procedures were the same as in study 1 except when noted otherwise. One chimpanzee was excluded from the subset of analyses involving comparisons of emotional and motivational responses to the safe outcome, as she never chose the safe option in the task.

### Results and discussion

#### Food preferences

For each species, we assigned the two most preferred food types as the good risk outcomes (chimpanzees; bread and banana; bonobos: apple and banana), and the two least preferred as the bad risk outcomes (chimpanzees: papaya and cucumber; bonobos: peanuts and lettuce), and the food type that was chosen intermediately as the safe option (chimpanzees: peanuts; bonobos: papaya). We then compared the two species' choices to ensure that they did not differ in their preferences for the food types assigned to each category. Results indicated that chimpanzees and bonobos did not differ in three of the food categories (p>0.15, n.s. for all cases, two-tailed t-test). However, bonobos exhibited greater preference for two of the possible risk outcomes: the most-preferred good risk outcome (chimpanzees chose bread on 80.0±2.6% of trials, whereas bonobos chose apple on 89.4±2.4% of trials; t_35_  = −2.25, p<0.05) and the least preferred bad risk outcome (chimpanzees chose cucumber on 5.7±1.5% of trials, whereas bonobos chose lettuce on 12.5±2.8% of trials; t_35_  = −2.05, p<0.05). Notably, both of these differences work against our predictions for these species' risk preferences, in that they suggest that bonobos actually prefer some of the food types produced by the risky option more than chimpanzees, and therefore should choose the risky option more often.

We next examined only those preference trials in which subjects chose between the intermediately-preferred food type (the safe option) and one of the possible risk outcomes, as this better reflected the choices apes actually faced in the risk task. Here, the two species did not differ in their relative preferences for any of the possible combinations (p>0.10 for all cases, n.s). Overall, these results indicate that we were able to select foods with approximately similar value for these species (with any deviations going against our main predictions), making it unlikely that the apes' behavior in risk task were the result of differences in food preferences.

#### Control trial performance

We used non-parametric statistics to assess control trial performance because data was highly skewed (apes approach ceiling levels of performance). Collapsing across all control trials, chimpanzees chose the correct option on 92.4±1.2% of trials, and bonobos chose correctly on 88.5±2.9%, with no species difference in overall performance (Mann Whitney U: z = −1.07, N_1_  = 24, N_2_  = 13, p = 0.32, n.s.). Moreover, both species performed highly on all individual control trial types (>80% correct), with no species differences for any individual control type (see Table 2). Importantly, these control trials involved the same inhibitory control, attentional, and memory demands as the main task, so it is unlikely that species differences in task comprehension can account for any differences in risk preferences.

**Table 2:** Performance of both species on control trials in study 2 (risky choice).

Control type	Chimpanzees	Bonobos	p-value
Inhibitory	95.8±2.4%	88.5±6.1%	0.36, n.s.
Comprehension-1	86.8±2.7%	82.1±.4.0%	0.39, n.s.
Comprehension-2	88.9±2.4%	88.5±4.0%	0.94, n.s.
Attention-1	97.9±.1.4%	92.3±.3.3%	0.27, n.s.
Attention-2	95.3±1.9%	94.2±4.2%	1.00, n.s.

Mean correct choices (± SE) across control trial types. Significance indicates whether the species differed in their performance in that control trial type (Mann Whitney U exact significance).

#### Risk preferences

**Figure 2:**
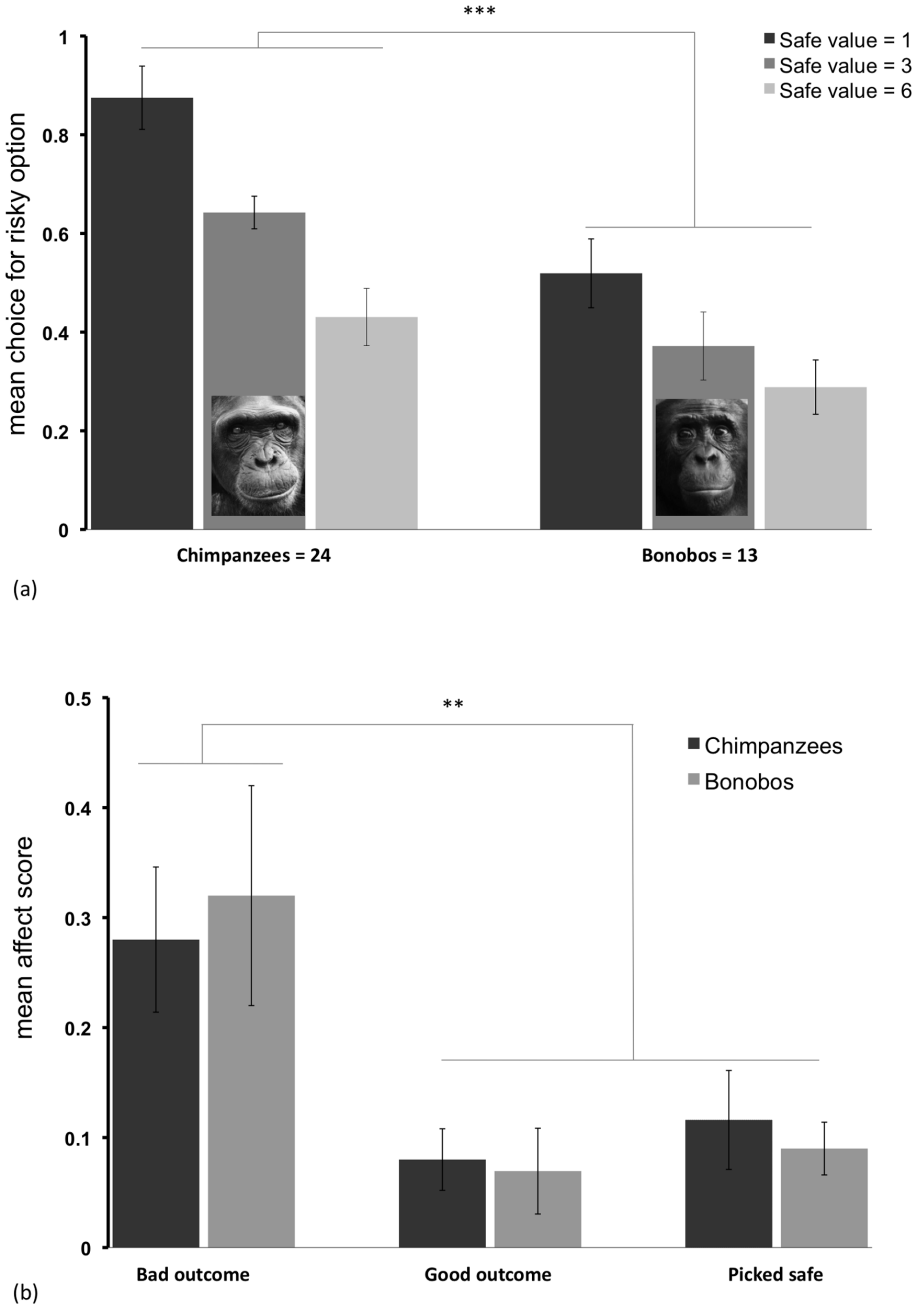
Risk preferences and affective responses in study 2. a) Chimpanzees and bonobos chose between a safe option that provided an intermediately-preferred food and a risky option that provided either a good outcome (highly-preferred food) or a bad outcome (non-preferred food). b) Composite affect scores following different choice outcomes (bad, good, or safe); a higher score indicates a more intense reaction with the production of more target behaviors (scratching, vocalizing, and banging). Error bars represent standard error of means.

Across all choice trials, chimpanzees chose the risky reward 64.9±4.1%, significantly above chance (one-sample t-test: t_23_  = 3.63, p<0.001, one-tailed). Bonobos, in contrast, chose the risky option on 39.3±5.3% of trials, below chance (one-sample t-test: t_12_  = −2.02, p<0.05, one-tailed). An initial analysis including condition as a factor indicated no difference between the low-variance and high-variance conditions (see SI), so all analyses reported here collapse across condition to reduce factors. A repeated-measures ANOVA revealed that chimpanzees chose the risky option more frequently than bonobos (F_1,35_  = 13.85, p = 0.001). In addition, apes chose the risky option less as value of the safe alternative increased (F_2,70_  = 47.84, p<0.001), as predicted. Finally, there was an interaction between safe value and species (F_2,70_  = 6.26, p<0.005); post-hoc tests revealed that chimpanzees chose the risky option more often than bonobos when the safe option offered one or three pieces of food, but not when the safe option offered six pieces (Tukey test, p<0.05 for all significant cases). This suggests that both species modulated their preferences across different safe option vales, and converged to similar levels of choice when the value of the safe option became large (see Figure 2A). These results align with previous findings that chimpanzees are more risk-prone than bonobos [22], [23], [24].

To exclude alternative explanations for this difference in risk preferences, we performed several additional analyses. First, there was no change in risk preferences within sessions (F_1,35_  = 0.014, p = 0.91, n.s.), and no species interactions, so different rates of satiation or learning cannot account for our results. Second, chimpanzees and bonobos did not differ from chance in the percentage of trials where they received the desirable outcome from the risky option (mean  = 48.4±1.5%; one-sample t-test: t_36_  = −1.02, p = 0.31, n.s.), nor did they differ from each other (t_35_  = −0.45, p = 0.66, n.s.). This indicates that the apes could not detect which outcome they would receive from the risky option before making their choice (e.g., by using some other cue such as smell), and both species experienced similar reward histories during the task. Finally, we looked at performance in the first trial of each condition. Chimpanzees preferred the risky option in both the low-variance (20/24 chose risk; binomial: p<0.005) and the high-variance conditions (19/24 chose risk; binomial: p<0.01), whereas bonobos showed no preference in either condition (low: 4/13 preferred risky; high: 9/13 preferred risky; binomial: p>0.25 in both cases). Thus, these species differed even before any learning via feedback in a given session could have occurred. Overall, this suggests these alternative possibilities cannot explain our main results.

#### Emotional and motivational responses

To assess the role of emotion in risky decisions, we first examined apes' responses after the outcome of their choice was revealed (e.g., good, bad, or safe outcomes). Specifically, we examined whether the apes had higher affect scores when they chose the risky option and received a bad outcome, compared to when they received a good outcome or chose the safe option (see Figure 2B, and Videos S3 and S4). Whereas apes had low affect scores after receiving the good outcome (mean score  = 0.08±0.02) or choosing safe (score  = 0.10±0.03), they had more intense negative responses following a bad outcome, with scores almost three times as high (score  = 0.29±0.05). Indeed, there was a significant effect of outcome on responses (Friedman test, n = 36, χ^ 2^(2)  = 23.51, p<0.001); pair-wise comparisons indicated that apes had higher scores after receiving bad outcomes compared to either good or safe outcomes (Wilcoxon signed-rank tests; n = 36 for all cases; bad versus good: z = −4.31, T+  = 25, 9 ties, p<0.005; bad versus safe: z = −3.84, T+  = 23, 7 ties, p<0.001), but there was no difference between responses to good and safe outcomes (p>0.45, n.s.). We next examined responses to different outcomes for each type of target behavior (see Table 1 for means). A comparison of responses to bad outcomes versus good or safe outcomes (here collapsing both categories to reduce factors given similar low rates of responses to safe and good outcomes) indicated that apes produced all target behaviors more following bad outcomes (n = 37 for all comparisons; *scratching*: z = −3.04, T+  = 19, 11 ties, p<0.005; *negative vocalization*: z = −3.21, T+  = 14, 22 ties, p<0.005; *banging*: z = −2.37, T+  = 7, 30 ties, p<0.05). Overall, these results indicate that apes show more negative emotional reactions when receiving undesired outcomes when making risky decisions.

We next examined whether these emotional responses mapped onto species- or individual-differences in risk preferences. First, both species showed more negative reactions in response to the bad outcome, compared to the good or safe outcome, when examined independently (chimpanzees: n = 24, z = −2.90, T+  = 15, 6 ties, p<0.005; bonobos: n = 13, z = −2.49, T+  = 10, 2 ties, p<0.05). A comparison of the two species' composite affect score in response to different outcomes revealed that there was no difference in their reactions to any category (good, bad, or safe; Wilcoxon signed-rank tests, p>0.5 for all comparisons). Moreover, there was no difference in how often they produced any particular target behavior (p>0.35 for all cases). As in study 1, we then calculated a difference score for each subject (their average affect score following a bad outcome, minus their score following a safe – and thus already known – outcome as a behavioral baseline). In chimpanzees, there was a trend for this index to be positively related to individual risk preferences (Spearman's ρ = 0.38, p = 0.07, two-tailed), but there was no relationship in bonobos (ρ = −0.28, p = 0.36, n.s.). Thus, these results demonstrated that both species showed negative responses to bad outcomes in the risk task. Taken with study 1's findings, this indicates that chimpanzees are not generally more reactive than bonobos across all contexts. However, the two species had similar responses to the different outcomes. There was only weak evidence for differences: chimpanzees with more negative reactions to bad risk outcomes tended to have a *greater* propensity to choose the risky option.

**Figure 3:**
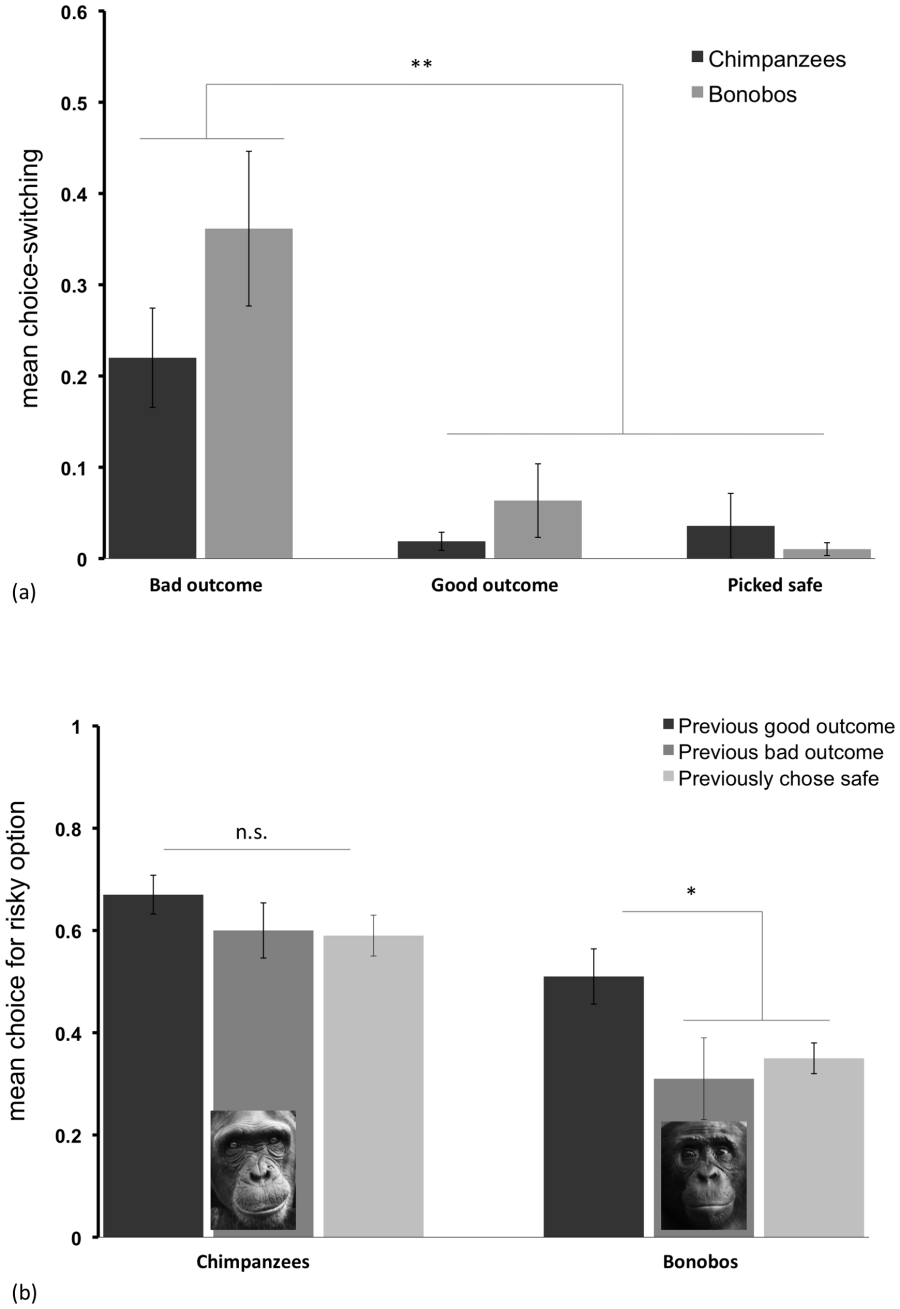
Choice switching and reward sensitivity in study 2. a) Chimpanzees and bonobos attempted to switch their choice in response to different outcomes in the risk task. b) The impact of previous trial outcome on current choice in chimpanzees and bonobos. Error bars represent standard error of mean.

As an additional measure of emotional responses in the risk task, we examined whether apes attempted to switch their choice after the experimenter revealed the outcome, a behavior that apes spontaneously exhibited in the task. That is, we examined whether apes immediately tried to choose to the alternative once they saw what they had received from their chosen option (they were never given the alternative in these instances). This type of response is consistent with counterfactual reasoning about what would have happened had one chosen differently, an important component of regret in humans [26], and other comparative studies have suggested that primates can engage in this type of counterfactual reasoning [75], [76]. Apes attempted to switch their choice on 27.1±4.6% of trials following bad outcomes, but rarely following good or safe outcomes (less than 4% of trials; see Figure 3A and Table 1). There was a significant effect of outcome on switching behavior (Friedman test, n = 36, χ^ 2^(2)  = 35.35, p<0.001); pair-wise comparisons revealed that apes attempted to switch more following bad outcomes (Wilcoxon signed-rank tests; n = 36 for all cases; bad versus good: z = −4.52, T+  = 26, 9 ties, p<0.001; bad versus safe: z = −4.37, T+  = 27, 6 ties, p<0.001), but there was no difference in switching in response to good and safe outcomes (p>0.95, n.s.). That is, apes selectively attempted to modify their choice only when they received a bad outcome. Indeed, both species showed more choice-switching in response to the bad outcome, compared to the good or safe outcome, when examined independently (chimpanzees: n = 24, z = −3.50, T+  = 17, 5 ties, p<0.001; bonobos: n = 13, z = −2.90, T+  = 10, 2 ties, p<0.005). Comparing the two species indicated no differences in their overall rate of switching attempts in response to any outcome (Mann-Whitney U, p>0.12 for all cases). Finally, we calculated individual index of choice switching (average switching following a bad outcome, minus switching following choices for safe as a behavioral baseline). This switching index correlated with risk preferences in bonobos (Spearman's ρ = −0.64, p<0.05) but not chimpanzees (Spearman's ρ = 0.14, p = 0.54, n.s.). That is, bonobos who were most like to attempt to switch their choice following undesirable outcomes showed the lowest propensity to choose the risky option overall, in line with results from humans relating high levels of regret to increased risk-aversion [26], [27], [31], [32], [77].

Lastly, to assess reward motivation we examined how the different risk outcomes impacted choices on the subsequent trial. We examined each species separately as they differed in overall risk preferences. Bonobos choose the risky option on 50.6±4.5% of trials following good risk outcomes, but on only 31.4±8.1% of trials follow bad risk outcomes and 35.3±3.7% following safe outcomes (see Figure 3B). A repeated-measures ANOVA revealed a main effect of previous outcome (F_2,24_  = 6.13, p<0.01), and post-hoc tests revealed that bonobos chose the risky option significantly more following good outcomes (Tukey tests, p<0.05 for all significant cases). Chimpanzees, however, were equally likely to choose the risky option regardless of previous outcome (F_2,44_  = 2.52, p>0.10, n.s). Finally, we calculated a reward sensitivity difference score (choices for risk following good outcomes, minus following bad outcomes). This index of reward sensitivity co-varied with overall risk preferences in bonobos (r_P_  = −0.71, p<0.01, two-tailed), but not chimpanzees (r_P_  = −0.24, p = 0.27, n.s). That is, bonobos who were more sensitive to disparities in the reward outcomes were less likely to choose the risky option overall. Overall, these results indicate that bonobos used a win-stay lose-shift strategy, preferring the risky option more following a good outcome than a bad outcome [78]. However, chimpanzees did not adjust their choices in response to the outcome they had received previously – despite using such a strategy in social contexts [79].

## General Discussion

Overall, our results indicate that chimpanzees and bonobos show affective and motivational responses when making decisions. In the temporal task, apes responded more negatively to waiting, exhibiting all three target behaviors more often following a choice for the delayed option. In the risk task they responded negatively to undesired outcomes, producing all three target behaviors more often when they chose the risky option and received the low-value payoff. In addition, apes selectively attempted to switch their choices following undesired outcomes. These results indicate that the types of emotional displays that apes exhibit in conspecific social interactions, such as negative vocalizations, are also exhibited in economic decision-making contexts. Overall, these results indicate decision-making in apes involves affective and motivational processes, similar to those seen in humans.

Some weaker evidence further suggests that these processes may be related to overall differences in choice preferences. In line with previous work [21], [22], [23], [24], the current studies indicted that chimpanzees and bonobos show divergent patterns of decision-making: chimpanzees were more patient and more risk-prone than were bonobos. Some aspects of the apes' responses mapped onto these differences in their preferences at the species- or individual- level. In the temporal choice task, the more patient chimpanzees showed more intense negative responses (with more emotional vocalizations specifically) while waiting than did bonobos. In the risk task, the most risk-averse bonobos showed the strongest reactions to disparities in reward outcome and made the most attempts to correct their choices following undesirable outcomes. Notably, these patterns of emotional responses in the two species did not always align with our original predictions (based on human studies) that bonobos might be more impatient and more risk-averse because they have strong negative reactions in response to waiting for rewards and receiving bad payoffs. In the temporal task, chimpanzees in fact exhibited more negative reactions to waiting than did the bonobos. In the risk task, chimpanzees and bonobos showed similar negative responses to bad outcomes (both in terms of the affect score measure and choice switching behaviors). However, these emotional responses had different impacts on the two species: whereas bonobos who often attempted to correct their choices (e.g., showed high levels of ‘regret’) were most risk-averse, there was no relationship in chimpanzees. Moreover, whereas bonobos modulated their current choices based on previous outcome, chimpanzees did not.

What can account for these patterns in the two species? In the risk task, there was some suggestion that differences in reward sensitivity may have been an important influence on the two species' behavior. In particular, chimpanzees may be so motivated to acquire the high-quality payoffs that they showed strong preferences for the risk option, despite pronounced negative response to receiving the bad outcome. That is, although both species reacted negatively to receiving the bad outcome, only bonobos actually modulated their subsequent choices in response to previous outcomes. Another possibility raised by result from the discounting task is that these species differ in their ability to regulate their emotional responses in some contexts. Human are able to exert cognitive control over their emotional responses, in particular by reappraising how they view events. These regulatory strategies are quite complex, and are thought to be lacking in some nonhuman models [5]. However, evidence from apes suggests that individuals may be able to engage in some relevant regulatory behaviors while waiting delays [80]. While previous studies have assessed how apes can use self-distraction to redirect attention, this type of paradigm could also be used to address whether chimpanzees and bonobos differ in their ability regulate their emotional responses when waiting. A final possibility is that although both species exhibited negative responses such as scratching and vocalizing, they actually differed in terms of the more specific emotion they experienced response to the two tasks. An analogous phenomenon occurs in chimpanzees' and bonobos' hormonal responses to food competition. While both species show similar behavioral responses with faced with competition with partners of various relative dominance status, these behaviors are supported by different underlying biological mechanisms: chimpanzees exhibit changes in testosterone in response to unequal-sharing situations, whereas bonobos exhibit changes in cortisol [81]. Thus, in the current set of studies, the underlying psychological experiences of the two species may have differed in a subtle way that our behavioral measures did not capture, because delineating fine-grained differences in emotions is difficult in animals who cannot provide self reports.

An important consideration for future research is whether these emotional responses play a causal role in ape decision-making, as they do in humans. The current results indicate that apes show emotional responses to decision-outcomes, and some of the evidence relating patterns of emotional responding to patterns of choice is also suggestive of the possibility that these processes causally influence choice strategies. However, future research should more directly investigate the causal role of emotions in ape decision-making. There are several approaches that could address this issue. First, studies that involve larger numbers of trials per subject would have more power to relate emotional reactions to subsequent choices on a trial-by-trial basis. Although we examined how previous outcome related to subsequent choice in the current risk task, larger number of trials would be needed to conduct similar trials involving emotional response data (given that such responses do not occur every trial). Second, studies that actively manipulate the apes' emotional state could examine whether such changes can shift strategies, much like studies involving mood induction in humans [34], [35], [36], [37]. Indeed, there is some suggestion that related psychological states play a causal role in ape behavior. In particular, chimpanzees and bonobos are more risk-prone following competitive interactions [24]. As some views suggest that competitive contexts alter emotional or motivation states in apes [82], [83], this indicates that manipulating the ape's emotional or motivational state might causally impact their patterns of decision-making. Finally, affect can influence human preferences via several different pathways – including immediate emotions at the time of choice and expectant emotions about what will happen in the future [1]. Physiological measures that assess the apes' emotional state immediately prior to choice [55], [84] could therefore be used assess the role of anticipatory emotions in these species' decision-making. This type of evidence will be critical to further test the hypothesis that emotion and motivation shape the divergent economic preferences exhibited by chimpanzees and bonobos at the mechanistic level.

Previous studies comparing temporal choices in chimpanzees and bonobos involved delay titration methods that identify a unique ‘indifference point’ for each individual [21], whereas the current study used a temporal task with set delays. Comparisons of their risk preferences have used quantitative variance in the amount of amounts received [22], variation in knowledge concerning the location of rewards provided by the risky option [23], and qualitative differences in food types as in the current study [24]. Although it is difficult to conclusively demonstrate species differences in behavior or cognitive skills, overall this set of results suggest that differences between the two species might be robust across different populations of apes and at least some variations in task structure. The current studies investigated the proximate-level factors (e.g., emotional and motivation responses) that may underlie this difference. At the ultimate evolutionary level, we have proposed that chimpanzees and bonobos may exhibit these divergent species-typical preferences due to differences in their wild feeding ecology. Relative to bonobos, who are thought to have evolved in more productive environments, chimpanzees on average face more seasonal food variability, more competition in food patches, and have less access to common fallback foods like terrestrial herbaceous vegetation [85], [86], [87], [88], [89], [90], [91]. Our evolutionary hypothesis is therefore that feeding ecology has shaped psychology in *Pan* such that chimpanzees are more willing than bonobos to accept ‘costs’ to obtain food – including in situations involving delays, travel time, effort, or risk – thus promoting adaptive patterns of decision-making in these species. Interestingly, chimpanzees and bonobos do not show differences in how they respond to ambiguity (or knowledge about probabilities of outcomes) when level of risk is equated [66], nor did they generally differ on most tasks in battery examining a wide range of cognitive skills [92]. This suggests that these species may exhibit targeted differences in only certain aspects of cognition and behavior that are related to differences in their socio-ecology [93].

These types of evolutionary analyses suggest that studies of apes may be important for addressing a major problem in the human cognitive sciences: characterizing functional systems supporting complex cognitive functions in the mind and the brain. Although investigations of chimpanzee and bonobo neurobiology are rare, largely due to important ethical considerations, non-invasive studies of these apes may provide a critical test for mapping function onto structure – especially for psychological features that may not be widely shared by common model species. For example, observed differences in the relative size and cytoarchitecture of orbitofrontal cortex of chimpanzees and bonobos [94] are consistent with this region's role in reward processing, emotional responses, and decision-making. Our results further predict a number of neurobiological differences between chimpanzees and bonobos: the anterior insula's role in risk-aversion [95] and the ventral striatum's role in both risky and intertemporal choice [95], [96] suggest that bonobos and chimpanzees may show divergence in these regions [97], [98]. More broadly, comparative analyses of brain evolution indicate that brain systems or networks can be characterized by examining whether given regions evolve together [99]. This type of evolutionary approach suggests that psychological capacities that evolve in tandem may act together to solve a given ecological or social problem. If the patterns of decision-making seen in *Pan* are an adaptive solution to their divergent natural ecologies, then changes in complex abilities such as decision-making may require joint selection on emotional and cognitive systems. The integration of such ultimate-level hypotheses into the human cognitive sciences can lead to an understanding of psychological and neurobiological systems in humans that is grounded in evolutionary function – what such systems are actually designed to do.

## Supporting Information

Figure S1 Setup for study 1 (temporal task). See SI Text for description. The human experimenter in this photograph has given written informed consent, as outlined in the PLOS consent form, to the publication of their photograph.
(TIFF)

Figure S2 Setup for study 2 (risk task). See SI Text for description. The human experimenter in this photograph has given written informed consent, as outlined in the PLOS consent form, to the publication of their photograph.
(JPG)

Figure S3 Setup for control trials in risk task. See SI Text for description. Food types pictured mirror those used with chimpanzees in the low-variance condition (banana is highly-preferred, cucumber is low-preferred, and peanuts are intermediately-preferred).
(JPG)

Table S1 Subject characteristics. Information on the species (C  =  chimpanzee, B  =  bonobo), sex (M  =  male, F  =  female), age (in years), and study participation for apes in both study 1 (temporal task) and study 2 (risk task).
(DOCX)

Text S1 Supplementary methods and results.
(DOCX)

Video S1 Bonobo temporal choice. The bonobo chooses the larger, delayed reward (3 pieces of food) over the smaller, immediate reward (1 piece of food). He scratches after his choice.
(MOV)

Video S2 Chimpanzee temporal choice. The chimpanzee chooses the larger, delayed reward (3 pieces of food) over the smaller, immediate reward (1 piece of food). He scratches, vocalizes, and bangs after his choice.
(MOV)

Video S3 Bonobo risky choice. The bonobo chooses the risky option (that provides either a piece of banana – a highly-preferred food – or a piece of lettuce – a non-preferred food – with equal probability) over a safe option that provided 6 pieces of an intermediately preferred food (papaya). After the experimenter reveals that he received a bad outcome, he attempts to switch his choice and scratches.
(MOV)

Video S4 Chimpanzee risky choice. The chimpanzee chooses the risky option (that provides either a piece of banana – a highly-preferred food – or a piece of cucumber – a non-preferred food – with equal probability) over a safe option that provided 6 pieces of an intermediately preferred food (peanuts). After the experimenter reveals that he received a bad outcome, he vocalizes and bangs.
(MOV)
