# Supplementary figures and images for: Chimpanzees and Bonobos Exhibit Emotional Responses to Decision Outcomes
Source: PLoS One. 2013 May 29;8(5):e63058. doi: 10.1371/journal.pone.0063058 (PMC3667125; doi:10.1371/journal.pone.0063058)

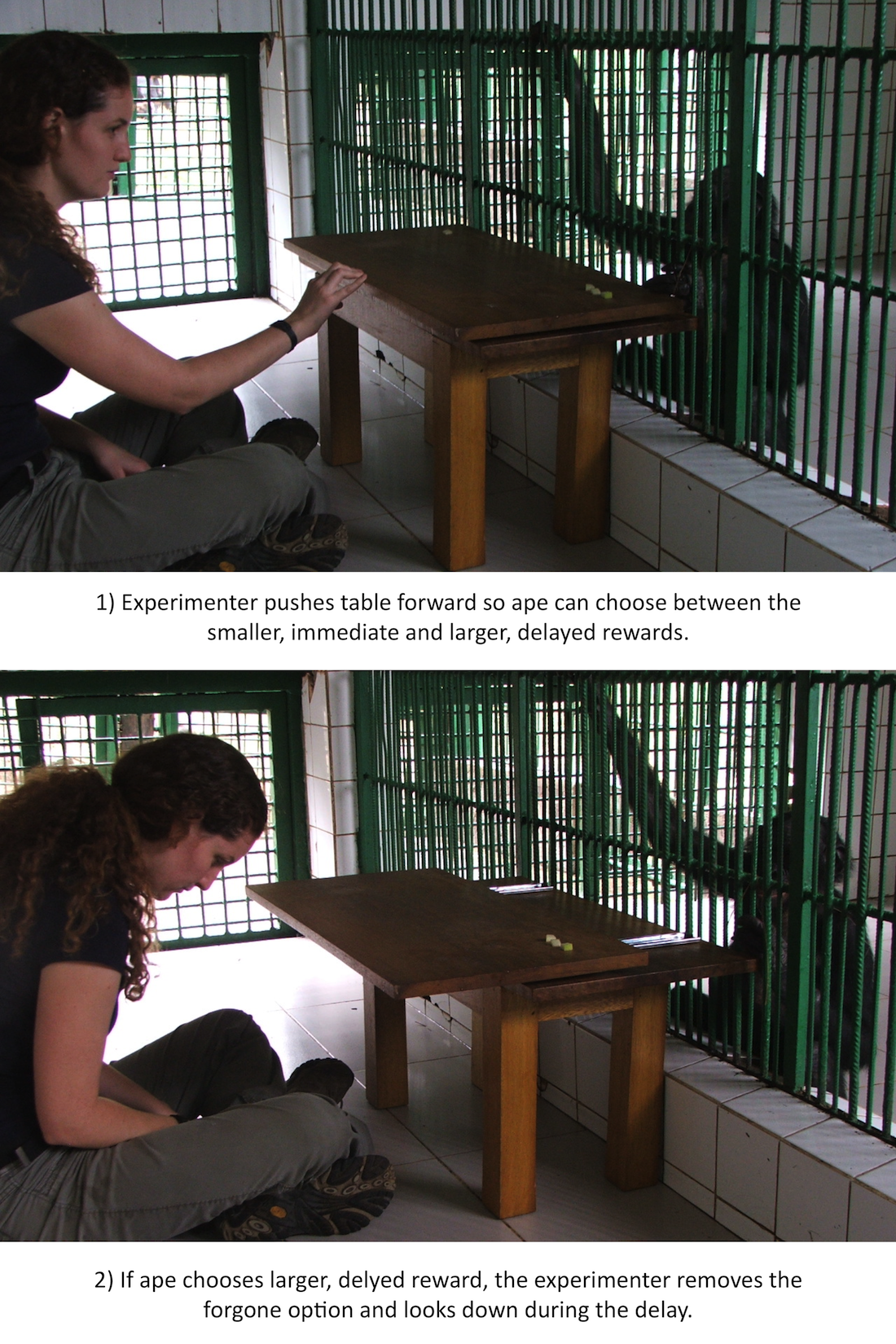

Supplement: Figure S1 — Setup for study 1 (temporal task). See SI Text for description. The human experimenter in this photograph has given written informed consent, as outlined in the PLOS consent form, to the publication of their photograph. (TIFF) [file pone.0063058.s001.tiff]

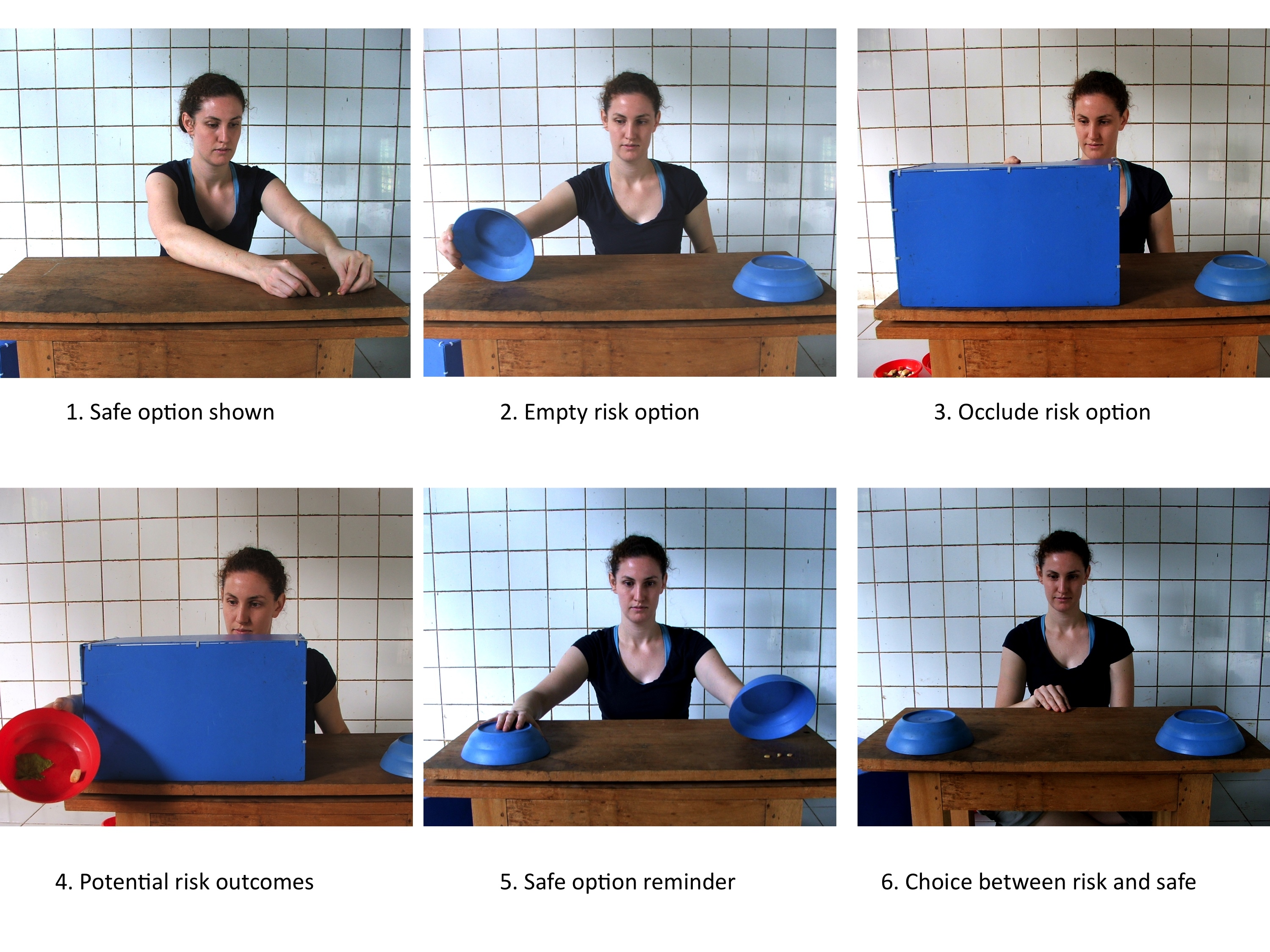

Supplement: Figure S2 — Setup for study 2 (risk task). See SI Text for description. The human experimenter in this photograph has given written informed consent, as outlined in the PLOS consent form, to the publication of their photograph. (JPG) [file pone.0063058.s002.jpg]

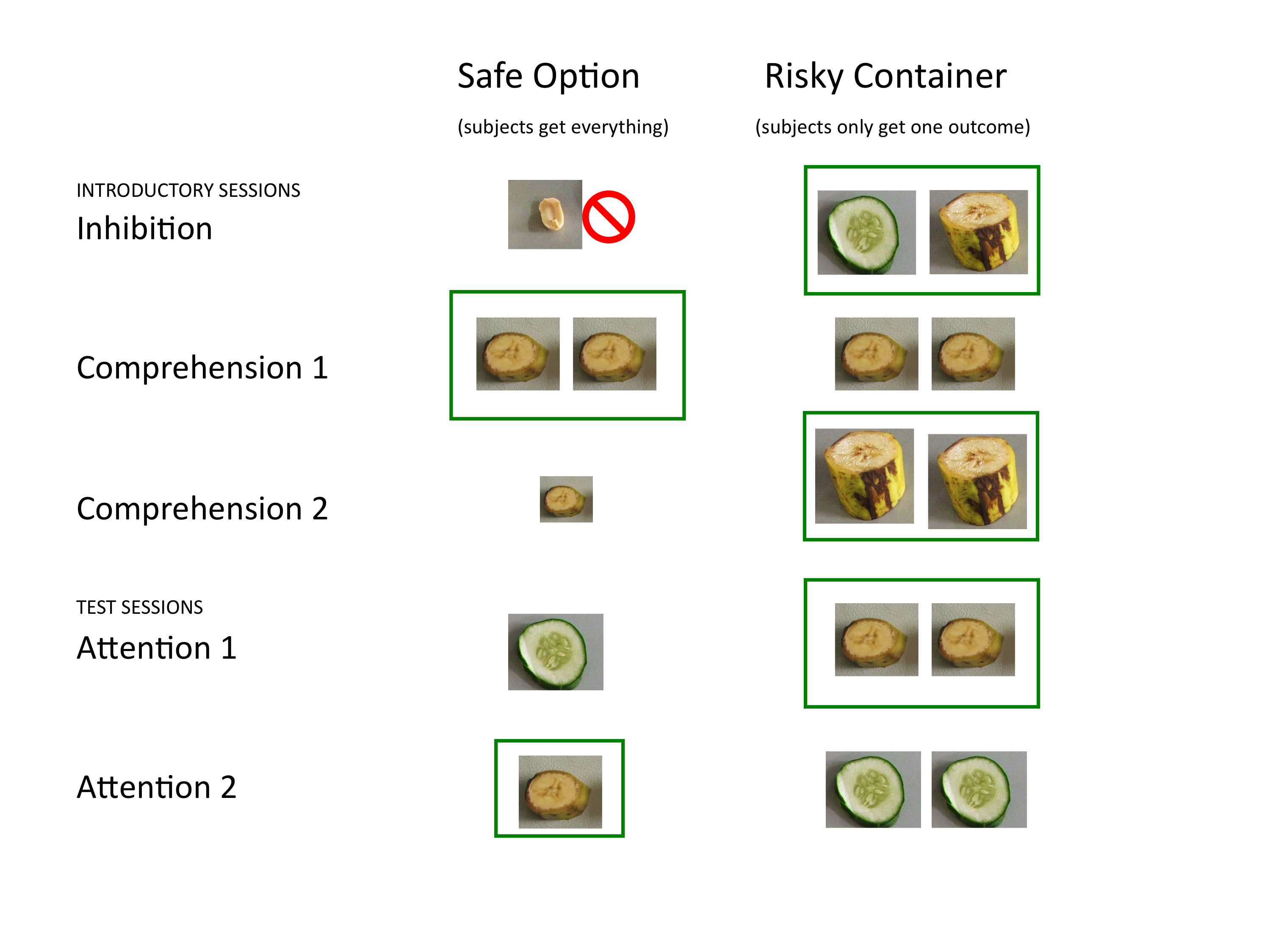

Supplement: Figure S3 — Setup for control trials in risk task. See SI Text for description. Food types pictured mirror those used with chimpanzees in the low-variance condition (banana is highly-preferred, cucumber is low-preferred, and peanuts are intermediately-preferred). (JPG) [file pone.0063058.s003.jpg]
