# Supplementary material for: Chimpanzees and Bonobos Exhibit Emotional Responses to Decision Outcomes
Source: PLoS One. 2013 May 29;8(5):e63058. doi: 10.1371/journal.pone.0063058 (PMC3667125; doi:10.1371/journal.pone.0063058)
Supplement: Table S1 — Subject characteristics. Information on the species (C = chimpanzee, B = bonobo), sex (M = male, F = female), age (in years), and study participation for apes in both study 1 (temporal task) and study 2 (risk task). (DOCX) [file pone.0063058.s004.docx]

| **Subject** | **Species** | **Sex** | **Age** | **Study Participation** |
| --- | --- | --- | --- | --- |
| Bayokele | C | F | 11 | 1, 2 |
| Bouetoussa | C | F | 8 | 1, 2 |
| Cherie | C | F | 14 | 1, 2 |
| Chimpie | C | M | 10 | 1, 2 |
| Christophe | C | M | 18 | 1, 2 |
| Elikia | C | M | 20 | 1, 2 |
| Kiki | C | F | 18 | 1, 2 |
| Kola | C | M | 11 | 1, 2 |
| Kondi | C | M | 9 | 1, 2 |
| Maya | C | M | 14 | 1, 2 |
| M'Bolo | C | F | 12 | 1, 2 |
| M'Boumbou | C | M | 19 | 1, 2 |
| N'Gouba | C | F | 10 | 1, 2 |
| Pembele | C | F | 16 | 1, 2 |
| Ramsay | C | F | 12 | 1, 2 |
| Silaho | C | F | 10 | 1, 2 |
| Sobele | C | M | 10 | 1, 2 |
| Tabonga | C | M | 9 | 1, 2 |
| Tchimaka | C | M | 9 | 1, 2 |
| Tiki | C | M | 7 | 1, 2 |
| Timi | C | M | 10 | 1, 2 |
| Ulemvouka | C | F | 8 | 2 (stopped participating in 1) |
| Wolo | C | M | 11 | 1, 2 |
| Yoko | C | M | 12 | 1, 2 |
| Api | B | M | 7 | 1, 2 |
| Beni | B | M | 10 | 1, 2 |
| Boende | B | M | 8 | 1, 2 |
| Dilolo | B | M | 7 | 1, 2 |
| Kalina | B | F | 10 | 1, 2 |
| Kasongo | B | M | 6 | 1, 2 |
| Kikongo | B | M | 7 | 1, 2 |
| Kikwit | B | M | 10 | 1, 2 |
| Kubulu | B | M | 6 | 1, 2 |
| Likasi | B | F | 7 | 1 (did not complete food preference in 2) |
| Lisala | B | F | 7 | 2 (stopped participating in 1) |
| Maniema | B | M | 7 | 1, 2 |
| Matadi | B | M | 8 | 1, 2 |
| Mbandaka | B | M | 7 | 1 (did not complete food preference in 2) |
| Mixa | B | M | 9 | 1, 2 |
| Nioki | B | F | 10 | 1, 2 |
