# Supplementary material for: Chimpanzees and Bonobos Exhibit Emotional Responses to Decision Outcomes
Source: PLoS One. 2013 May 29;8(5):e63058. doi: 10.1371/journal.pone.0063058 (PMC3667125; doi:10.1371/journal.pone.0063058)
Supplement: Text S1 — Supplementary methods and results. (DOCX) [file pone.0063058.s005.docx]

**Supporting Information**

**Supplementary Methods and Analyses**

Chimpanzees and bonobos exhibit emotional responses to decision outcomes

Alexandra G. Rosati & Brian Hare

**Study 1: Temporal preferences**

*Setup for temporal task:* As shown in Figure S1, the procedure was as follows. (1) The experimenter pushed a sliding table forward so that subjects could choose between a smaller, immediate reward and three delayed pieces. (2) If subjects choose the large reward, the experimenter removed the forgone option and left the chosen option on the table. During the delay the experimenter looked down and did not interact with the subject until the delay concluded and they gave the ape the reward.

*Age and sex:* We examined whether age had any impact on apes’ temporal preferences. We first correlated overall choice for the large, delayed reward with the estimated age for each subject. There was no effect in either species (bonobos: r_P_ = 0.25, p = 0.36, n.s.; chimpanzees: r_P_ = 0.03, p = 0.91, n.s.). Because only chimpanzees had enough numbers of both male and female subjects to compare statistically, we only examined sex and discounting preferences in chimpanzees, and there was no effect (t_21_ = -0.60, p = 0.56, n.s.).

**Study 2: Risk preferences**

*Setup in risk task:* As shown in Figure S2, the procedure in this study was as follows. (1) The experimenter placed the safe option on the table in view of the subject, and then covered it with a bowl. (2) The experimenter showed the subject the empty risk bowl and (3) occluded it. (4) The experimenter showed the possible risky outcomes (half good, half bad) for that trial. Behind the occluder, the experimenter then placed one of the outcomes under the risk bowl. (5) After removing the occluder, the experimenter reminded the subject of the safe alternative’s value. (6) Finally, the experimenter pushed the sliding table forward so the cups were in reach of the subject to choose.

*Control trial types:* As shown in Figure S3, apes completed five types of control trials where the type, size, and quantity of food varied from that used in choice trials, to assess their comprehension of the task. If they choose the risky option, subjects always received only one of the possible outcomes they saw in the risk outcome container; both potential outcomes are pictured here. For each trial type, the correct choice is marked with a box. In *inhibition trials,* the procedure was identical to that in normal risky choice trials, but in a final step the experimenter removed the food from under the safe option (should choose risky). In *comprehension-1 trials*, the safe option provided two pieces of a preferred food, and subjects saw two identical pieces in the risk outcome container (should choose safe). In *comprehension-2 trials*, the safe option provided a small piece of a preferred food, and the risky outcome container contained two larger pieces of the preferred food (should choose risky). In *attention-1* trials, the safe option provided one piece of a preferred food, and subjects saw two pieces of a non-preferred food in the risky outcome container (should choose risky). In *attention-2 trials,* the food types were reversed (should choose safe).

*Low- and high-variance conditions:* We first conducted an analysis of the risk choices including both condition (high versus low variance) and safe value (1, 3, or 6) as within-subject factors and species as a between-subject factor. Results revealed main effects of species (F_1,35_ = 13.99, p = 0.001] and safe value [F_2,70_ = 48.04, p < 0.001) but no effect of condition (F_1,35_ = 0.57, p = 0.46, n.s.). There was a trend for a significant interaction between condition and species (F_1,35_ = 3.95, p = 0.055). We conducted post-hoc analyses to investigate this trend, which indicated that while neither species showed an effect of condition, chimpanzees in the high-variance condition were not significantly different from bonobos in the low-variance condition (Tukey test, p < 0.01 for all significant cases). As this was not relevant to our main hypotheses, and there were no relevant differences in responses to the low- and high-variance conditions, we consequently collapsed across condition for all analyses reported in the main text to reduce factors in other analyses.

*Age and sex:* We examined whether there were any effects of age or sex on risk preferences. As in the temporal choice study, we first correlated age with overall preferences for the risky option in both species. There was no correlation in either species (chimpanzees: r_P_ = -0.14, p = 0.50, n.s.; bonobos: r_P_ = -0.35, p = 0.24, n.s.). As there were only enough individuals of both sexes to statistically compare in chimpanzees, we examined the impact of sex in this species, but found no effect (t_22_ = 0.18, p = 0.43, n.s.).
